# Temporal association of sNfL and gad‐enhancing lesions in multiple sclerosis

**DOI:** 10.1002/acn3.51060

**Published:** 2020-05-25

**Authors:** Mattia Rosso, Cindy T. Gonzalez, Brian C. Healy, Shrishti Saxena, Anu Paul, Kjetil Bjornevik, Jens Kuhle, Pascal Benkert, David Leppert, Charles Guttmann, Rohit Bakshi, Howard L. Weiner, Tanuja Chitnis

**Affiliations:** ^1^ Harvard Medical School Boston Massachusetts 02115 USA; ^2^ Ann Romney Center for Neurologic Disease Harvard Medical School Boston Massachusetts 02115 USA; ^3^ Massachusetts General Hospital Biostatistics Center Boston Massachusetts USA; ^4^ Department on Nutrition Harvard T.H. Chan School of Public Health Boston Massachusetts USA; ^5^ Departments of Medicine, Biomedicine and Clinical Research Neurologic Clinic and Policlinic University Hospital Basel University of Basel Basel Switzerland; ^6^ Department of Clinical Research Clinical Trial Unit University Hospital Basel University of Basel Basel Switzerland; ^7^ Department of Neurology Partners Multiple Sclerosis Center Brigham and Women's Hospital Boston Massachusetts USA

## Abstract

**Objective:**

Multiple sclerosis (MS) is an autoimmune demyelinating disorder, which is characterized by relapses and remissions. Serum neurofilament light chain (sNfL) is an emerging biomarker of disease activity but its clinical use is still limited. In this study, we aim to characterize the temporal association between sNfL and new clinical relapses and new gadolinium‐enhancing (Gd+) lesions.

**Methods:**

Annual sNfL levels were measured with a single‐molecule array (SIMOA) assay in 94 patients with MS enrolled in the Comprehensive Longitudinal Investigation of Multiple Sclerosis at the Brigham and Women’s Hospital (CLIMB) study. We used a multivariable linear mixed‐effects model to test the temporal association of sNfL with clinical relapses and/or new Gd+ lesions. We adjusted this model for age, disease duration, sex, and disease‐modifying therapies (DMTs) use.

**Results:**

In the 3 months after a Gd+ lesion, we observed an average 35% elevation in sNfL (*P* < 0.0001) compared to remission samples. We also observed an average 32.3% elevation in sNfL at the time of or prior to a Gd+ lesion (*P* = 0.002) compared to remission. We observed a significant elevation in sNfL after a clinical relapse only when associated with a Gd+ lesion.

**Interpretation:**

Our findings support sNfL as a marker of clinical relapses and Gd+ lesions. sNfL peaks in a 3‐month window around Gd+ lesions. sNfL shows promise as a biomarker of neurological inflammation and possibly of simultaneous Gd+ lesions during a clinical relapse.

## Introduction

Multiple sclerosis (MS) is a chronic autoimmune disorder of the central nervous system (CNS), which presents with demyelination and axonal loss.^1^ MS has a chronic course, which starts in young adulthood and results in disability accrual over time. Most patients present with a relapsing‐remitting (RRMS) course, which is characterized by clinical relapses followed by remissions. Clinical relapses may affect different parts of the CNS, including the optic nerve, the spinal cord, the cerebrum, the cerebellum, and the brainstem. Clinical relapses are due to acute CNS inflammation, which may also be detected as new gadolinium‐enhancing lesion (Gd+) on MRI scans.^2^, ^3^ The correct assessment of MS relapses is crucial since higher relapse rates have been shown to result in a greater disability burden and overall worse prognosis.^4^, ^5^, ^6^, ^7^

While Gd+ lesions provide a direct correlate of CNS inflammation, the use of MRI scans is not available to all centers and is limited by long scanning times and high costs. Molecular biomarkers could be a cheaper and more convenient alternative to MRI scans. Recently, the axonal protein neurofilament light chain (NfL) has been proposed as a possible biomarker of disease activity in MS.^8^, ^9^, ^10^, ^11^, ^12^, ^13^, ^14^, ^15^ NfL is a class IV intermediate filament, which makes up the scaffolding of axons and facilitates structural stability and signal conduction in neurons.^16^, ^17^

NfL was first detected in the cerebrospinal fluid (CSF) but newer assays (e.g., the single‐molecule array‐SIMOA) with high sensitivity can reliably measure serum NfL (sNfL) levels.^18^, ^19^, ^20^, ^21^, ^22^, ^23^, ^24^ sNfL and CSF NfL alike have been shown to correlate with clinical relapses, MRI lesions, disability accrual (expanded disease status scale‐ EDSS), and brain atrophy.^21^, ^24^, ^25^, ^26^, ^27^, ^28^ Although these results are promising, literature has shown a high degree of heterogeneity in terms of reported NfL levels and study methods. Furthermore, the duration of sNfL elevation after disease activity is currently unknown, as are the dynamics of sNfL before disease activity. This information is key to determine the reference ranges for sNfL levels in patients with MS, as well as the time window of sNfL elevation.^22^

The goal of this study was to assess the relationship between sNfL and acute inflammatory disease activity in order to inform the use of sNfL in the clinical setting. We are specifically interested in the temporal dynamics of sNfL associated with clinical relapses compared to Gd+ lesions, which we assessed in separate analyses. Furthermore, we grouped the serum samples according to their association with prior and future disease activity.

## Methods

### Subjects

We selected 94 MS patients enrolled in the Comprehensive Longitudinal Investigation of MS at the Brigham and Women’s Hospital (CLIMB).^29^ The CLIMB study is a longitudinal study with more than 2100 patients enrolled since 2000. The patients included in this study (1) were enrolled in the quality of life (QOL) subgroup of the CLIMB study; (2) had a diagnosis of MS according to the 2010 McDonald criteria at last visit^30^; (3) had an available blood sample in the first 5 years of their first MS symptom; (4) had at least eight annual blood draws from the first collection to year 10; and (5) provided consent for sample sharing. The enrolled patients had biannual clinical visits and annual MRI scans according to our standardized protocol. All relevant clinical details were entered into the relational database according to the standardized protocol of the Partners MS Center.

Clinical relapses were characterized by the treating neurologist either at the time of a clinical relapse or during a subsequent clinical visit. Clinical information on relapses included the relapse onset date, relapse location, signs and symptoms, and relapse severity according to NINDS‐CDE criteria (https://www.commondataelements.ninds.nih.gov/MS.aspx#tab=Data_Standards).

Relapse severity and location were rated at the time of the clinical relapse or retrospectively. As per the standardized protocol, all relevant clinical information was then validated by a trained fellow who reviewed patient charts and MRI reports. All challenging cases were reviewed with an expert neurologist (TC). We excluded all serum samples associated with clinical relapses or questionable MRI findings.

### Patient consent

Informed consent was obtained from patients in an ongoing observational cohort study of MS patients at the Partners MS Center (CLIMB study). Institutional Review Board (IRB) approval was granted by the Partners Human Research Committee, and participants provided written informed consent for participation.

### sNfL measurements

Serum samples were collected during annual clinical visits and were stored at −80°C according to standardized procedures. The samples were shipped from Boston, Massachusetts to Basel, Switzerland where sNfL levels were measured with a SIMOA assay.^21^ Quality control was ensured during shipping; the samples were stored in a temperature‐controlled container. Additional details are included in our previous study on the same cohort.^31^

### Relapse groups

**Figure 1:**
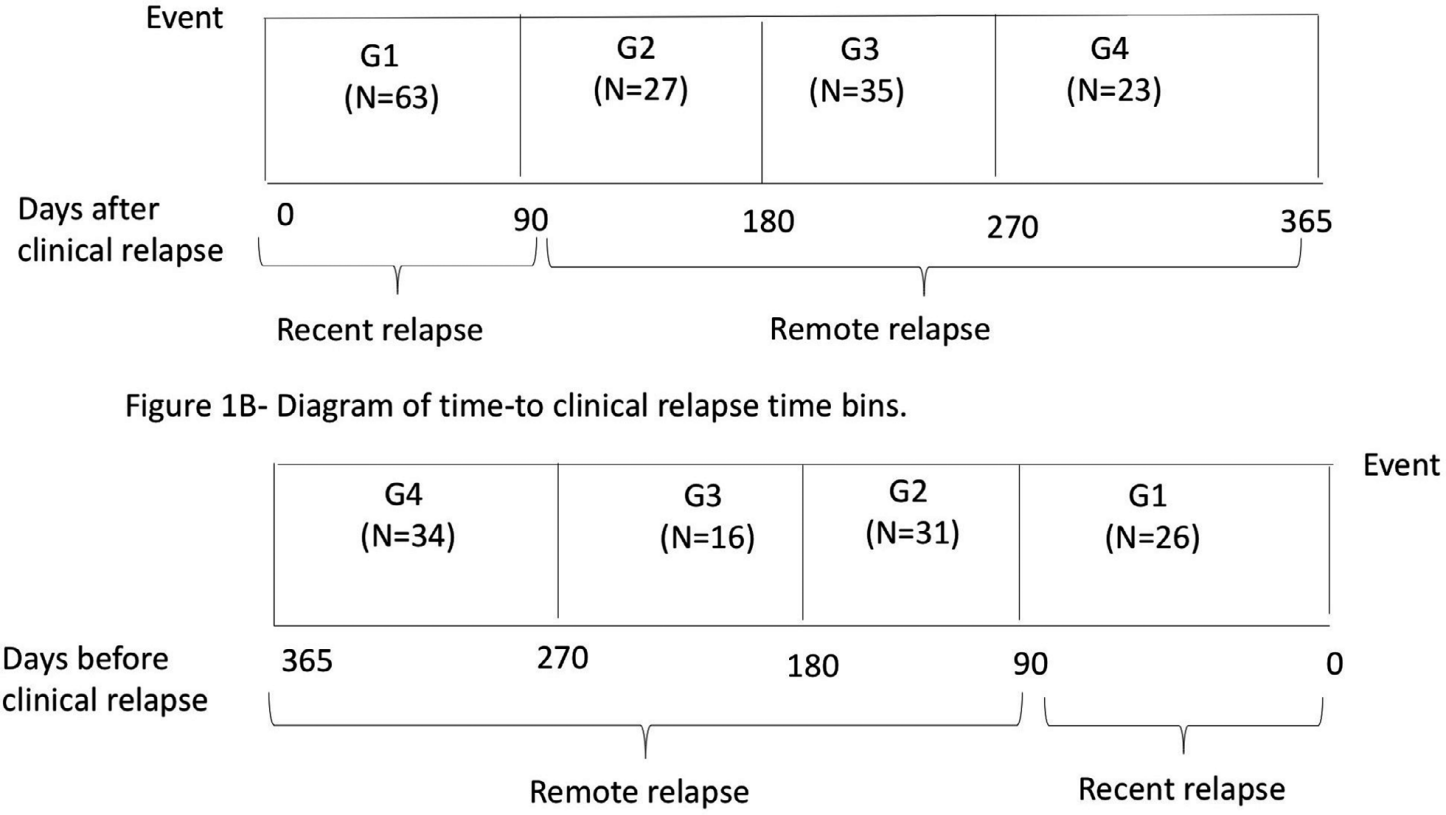
Diagram of time from/to disease activity. G1/2/3/4 = Group 1/2/3/4; N = number of samples in each time interval for clinical attack samples.

The primary goal of this study was to assess sNfL as a predictive and diagnostic marker of disease activity. We performed separate analyses for clinical relapses and Gd+ lesions. We also accounted for the time of disease activity relative to the time of blood samples (i.e., before a blood sample in Fig. 1A, and after a blood sample in Fig. 1B). We grouped the samples into discrete bins by the time from/to a clinical relapse. Figure 1 shows the time bins for both analyses, including (1) 0–90 days from the sample (G1), (2) 91–180 days from the sample (G2), (3) 181–270 days from the sample (G3), and (4) 271–365 days from the sample (G4). We also assessed a 0–30 days bin and 31–90 days bin, which did not appear to be more informative compared to a 0–90 days bin. In additional analyses, we classified subjects into recent clinical relapses/Gd+ lesions (G1 only) and remote clinical relapses/Gd+ lesions (G2–4) (Fig. 1).

We assessed the location and severity of clinical relapses in additional analyses. The severity profile was ranked as mild, moderate, or severe as rated by the treating neurologist. The treating neurologist also reported the location of the clinical relapse as brainstem–cerebellum, cerebrum, optic nerve, spinal cord, or any combination of these locations. Both analyses were stratified according to the time of the clinical relapses (recent vs. remote).

### MRI groups

We studied the time dynamics of sNfL before and after new Gd+ lesions on T1 MRI scans. We used brain MRI acquisition protocols using 1.5 T and 3 T units. The sequences were optimized in contrast to depict the brain–CSF interface and white matter lesions. We used T1‐weighted imaging sequences to evaluate the presence of Gd+ lesions in the proximity of MS lesions. The temporal dynamics of sNfL were assessed in relation to time since or from MRI lesions: (1) 0–90 days (G1), (2) 91–180 days (G2), (3) 181–270 days (G3), and (4) 271–365 days (G4).

### Reference groups

The reference group (remission group) comprised of samples from patients who had not experienced clinical relapses or Gd+ lesions in the year before or after they provided the blood sample. The time‐to‐disease activity analyses (i.e., to clinical relapse or to Gd+ lesion) included a reference group where the serum sample was not associated with any clinical relapse or Gd+ lesions in the 365 days after the time of sampling. Likewise, the time‐from‐disease activity analyses (i.e., from clinical relapse or from Gd+ lesions) included a reference group where the serum sample was not associated with any clinical relapse or Gd+ lesions during 365 days before the time of sampling.

### Statistical analysis

We analyzed the baseline clinical and demographic characteristics of our subjects. Counts and percentages were reported for all categorical variables. We reported median and interquartile range (IQR) for annualized relapse rate (ARR) for EDSS scores. For all other continuous variables, mean and standard deviation (SD) were used. We applied a linear mixed‐effects regression model with a random intercept to assess the log‐transformed sNfL levels across different time bins. We performed separate analyses for samples taken before and after a clinical relapse/Gd+ lesion. We also adjusted the analysis for age, disease duration, disease‐modifying therapy, sex, and use of DMTs and reported unadjusted and adjusted results. We have used LOcally Estimated Scatterplot Smoothing (LOESS) to show the trend of serum NfL over time. We performed a subanalysis where we analyzed clinical relapses and Gd+ lesions in conjunction. We formed nine groups of patients according to these labels.

Then, we analyzed the association between sNfL and the location of clinical relapses by classifying sNfL levels according to both severity and time interval, which resulted in seven groups (recent/mild, recent/moderate, recent/severe, remote/mild, remote/moderate, remote/severe, no clinical relapse). We compared these groups with linear mixed‐effects model as above. In these analyses, we included a reference group that consisted of serum samples from patients in continued remission for at least 365 days.

To assess the association between clinical relapse location and sNfL level, we grouped our cohort according to the time of the relapse (recent: within 0–90 days of the relapse; remote: within 91–365 days of the relapse) and to the location of the relapse as reported by the clinician. sNfL measurements were thus grouped into 13 groups, which included recent/brainstem–cerebellum, recent/cerebrum, recent/combined location, recent/optic nerve, recent/spinal cord, and recent/unknown location, remote/brainstem‐cerebellum, remote/cerebrum, remote/combined location, remote/optic nerve, remote/spinal cord, and remote/unknown location, no clinical relapse. The rationale for this grouping was the same as in the severity analysis– using a no‐relapse cohort as the reference group.

We assessed the differences among these groups using a mixed‐effects linear regression model as above. Analyses were performed using the Statistical Analysis System (SAS) 9.4 (Cary, NC) and R Studio 1.1.456 (R Studio Inc.).

## Results

### Subjects and serum samples

Table 1 shows the baseline demographic and clinical characteristics of our cohort (*n* = 94). The majority of patients were female with a mean age was 37 years with an overall disease duration of 2.3 years (Table 1). Most patients were treated with disease‐modifying therapies (DMTs), and interferon beta‐1a and glatiramer acetate were the two most commonly used therapies (36.7% and 27.5% respectively). Annual serum samples from these patients were characterized according to their association with clinical relapses, Gd+ lesions, or continued remission.

**Table 1:** Demographics of the patient population.

Characteristics	MS patients (*n* = 94)
Sex, *n* (%)	
Female	69 (73%)
Male	25 (27%)
Race, *n* (%)	
White	90 (96%)
African American	1 (1%)
Unknown	2 (2%)
More than one race	1 (1%)
Age, years at baseline (mean, SD)	37.4 ± 8.9
Disease duration, years at baseline (mean, SD)	2.3 ± 1.4
EDSS at baseline (median, interquartile range)^1^	1.0 (0–2.0)
DMTs at the time of sample collection^2^	
Abatacept	1 (0.2%)
Basiliximab	1 (0.2%)
Cyclophosphamide	8 (1.3%)
Daclizumab	4 (0.7%)
Dimethyl fumarate	9 (1.5%)
Fingolimod	13 (2.1%)
Glatiramer acetate	166 (27%)
Interferon beta‐1a	224 (36.4%)
Interferon beta‐1b	29 (4.7%)
Methotrexate	2 (0.3%)
Mycophenolate mofetil	10 (1.6%)
Natalizumab	39 (6.3%)
No DMT	106 (17.2%)
Pegylated interferon beta‐1a	1 (0.2%)

DMT, disease‐modifying therapy; EDSS, expanded disability status scale; MS, multiple sclerosis; *n*, patient count; SD, standard deviation.

^1^ Patients did not have an EDSS value and were excluded.

^2^ Patients can be counted for multiple times due to being on different DMTs at the time when sample was collected.

### sNfL levels after clinical relapse

**Figure 2:**
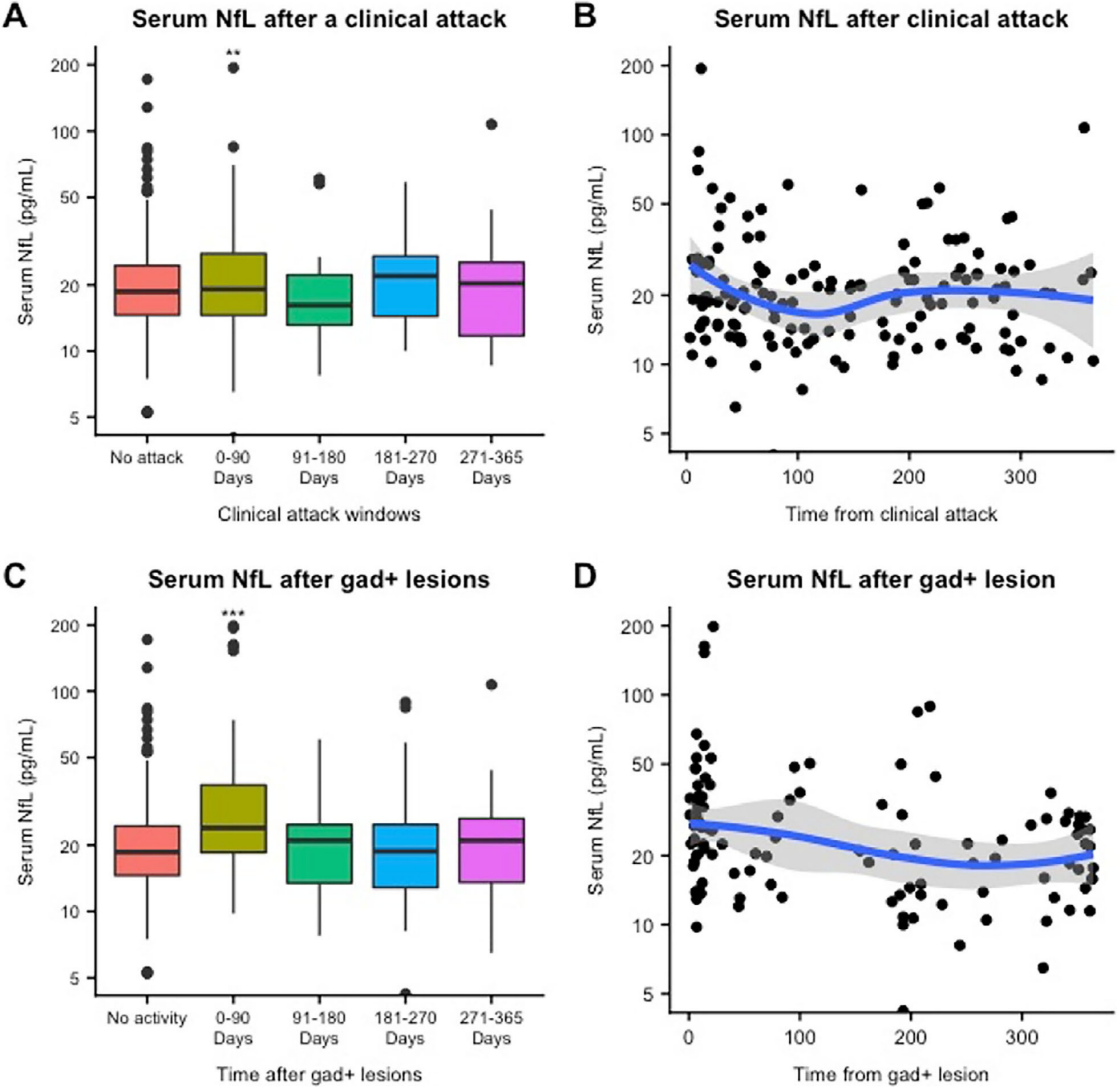
Serum NfL after disease activity. gad + lesion = gadolinium‐enhancing lesion; NfL = neurofilament light chain. **P ≤ 0.001, ***P ≤ 0.0001

When we investigated sNfL levels after a clinical relapse, we observed that sNfL levels were 20.9% higher within 90 days of a clinical relapse than in remission samples (95% CI: 7.3%–35%, *P* = 0.001). In all time bins of the 90‐day window, there was no difference between the remission and postclinical relapse groups. (Fig. 2A; Table 2).

**Table 2:** sNfL dynamics after acute disease activity in 3‐month increments.

Time bins	Clinical relapse				Acute Gd+ MRI lesion (gadolinium‐enhancing)			
	Unadjusted		Adjusted^1^		Unadjusted		Adjusted^1^	
	Delta sNfL^2^	*P* value	Delta sNfL^2^	*P* value	Delta sNfL^2^	*P* value	Delta sNfL^2^	*P* value
No disease activity (reference group) (*n* = 403)	Ref.	–	Ref.	–	Ref.	–	Ref.	–
0–90 days (clinical:*n* = 63; MRI:*n* = 93)	19.7% (7.2% to 33.6%)	**0.002** *	20.9% (7.3% to 35%)	**0.001** *	35% (23.4% to 47.7%)	**<0.0001** *	35% (23.4% to 47.7%)	**<0.0001** *
91–180 days (clinical:*n* = 27; MRI:*n* = 33)	–3.9% (–18.1% to 12.7%)	0.62	–3% (–17.3% to 12.7%)	0.66	10.5% (–5.8% to 28.4%)	0.22	10.5% (–5.8% to 28.4%)	0.23
181–270 days (clinical:*n* = 35; MRI:*n* = 49)	12.7% (–2% to 29.7%)	0.09	13.9% (–1% to 31%)	0.08	3% (–9.5% to 17.4%)	0.67	2% (–10.4% to 16.2%)	0.74
271–365 days (clinical:*n* = 23; MRI:*n* = 44)	–2.0% (–17.3% to 17.4%)	0.86	0.9% (–15.6 to 19.7%)	0.92	7.3% (–6.8% to 22.1%)	0.33	7.3% (–6.8% to 23.4%)	0.32

*n*, number of samples, sNfL, serum neurofilament light chain.

^1^ Analysis adjusted for age, disease duration, disease‐modifying therapy, sex.

^2^ Results are reported as percentage difference in sNfL, with 95% confidence interval.

* Significant *P*‐values are reported in bold text.

### sNfL levels after Gd+ lesion

When we analyzed sNfL levels after Gd+ lesions, we observed that sNfL levels were significantly higher within 90 days after a Gd+ lesion compared to remission samples (estimated percentage increase: 35%, 95% CI: 23.4%–47.7%, *P* < 0.0001). (Fig. 2C; Table 2) After 90 days, there was no difference between the remission and post‐ Gd+ lesion groups. Recent Gd+ lesions were associated with a significant percentage increase in sNfL irrespective of the presence of a clinical relapse (i.e., in samples with no associated clinical relapse in the prior 365 days, with an associated recent clinical relapse, and with an associated remote clinical relapse). (Table 3).

**Table 3:** sNfL dynamics after acute disease activity as a continuous variable.

Clinical groups	MRI groups	Delta sNfL—unadjusted^2^	Delta sNfL—adjusted^1^, ^2^
No clinical relapse	No Gd+ lesion (*n* = 403)	Ref.	Ref.
	Recent Gd+ lesion^3^ (*n* = 57)	44.8% (33.6%–64.9%) ***P* < 0.0001** *	44.8% (28.4%–64.9%) ***P* < 0.0001** *
	Remote Gd+ lesion^4^ (*n* = 34)	7.3% (–7.7%–24.6%) *P* = 0.37	8.3% (–6.8%–25.9%) *P* = 0.33
Recent clinical relapse^5^	No Gd+ lesion (*n* = 37)	2% (–11.3%–18.5%) *P* = 0.74	2% (–11.3%–18.5%) *P* = 0.75
	Recent Gd+ lesion^3^ (*n* = 19)	58.4% (29.7%–93.5%) ***P* < 0.0001** *	60% (29.7%–95.4%) ***P* < 0.0001** *
	Remote Gd+ lesion^4^ (*n* = 7)	36.3% (–0.7%–87.8%) *P* = 0.06	37.7% (0.1%–89.6%) *P* = 0.05
Remote clinical relapse^6^	No Gd+ lesion (*n* = 70)	0.4% (–10.4%–11.6%) *P* = 0.95	0.6% (–9.5%–12.7%) *P* = 0.91
	Recent Gd+ lesion^3^ (*n* = 17)	78.6% (44.8%–120.3%) ***P* < 0.0001** *	82.2% (47.7%–127%) ***P* < 0.0001** *
	Remote Gd+ lesion^4^ (*n* = 15)	22.1% (–2%–52.2%) *P* = 0.08	25.9% (–0.1%–58.4%) *P* = 0.05

*n* = number of samples; sNfL, serum neurofilament light chain.

^1^ Analysis adjusted for age, disease duration, disease‐modifying therapy, sex.

^2^ Results are reported as percentage difference in serum NfL, with 95% confidence interval and *P*‐values.

^3^ Recent Gd+ lesion = serum sample within 0–90 from Gd+ lesion.

^4^ Remote Gd+ lesion = serum sample within 91–365 from Gd+ lesion.

^5^ Recent clinical relapse = serum sample within 0–90 from clinical relapse.

^6^ Remote clinical relapse = serum sample within 91–365 from clinical relapse.

* Significant *P*‐values are reported in bold text.

### sNfL levels before clinical relapse

When we analyzed sNfL levels before a clinical relapse, we failed to observe any significant difference in samples taken prior to a clinical relapse compared to remission samples (Table 3). However, in a subanalysis (Table 3), we found a significant increase in sNfL only in relapse samples taken at the time of Gd+ lesion (estimated increase = 60%, 95% CI: 29.7%–95.4%; *P* < 0.0001). sNfL levels were also significantly elevated in serum samples taken 181–270 days before a clinical relapse (estimated increase = 31%, 95% CI: 6.2%–61.6%, *P* = 0.01). In an additional subanalysis of combined clinical relapses and Gd+ lesions, we only found an association between remote clinical relapses (i.e., between 91 and 365 days after the event) and sNfL in the presence of Gd+ lesions (estimated percentage elevation = 180.1%; 95% CI: 99.4%–289.6%; *P* < 0.0001**)**. (Table 5).

### sNfL levels before Gd+ lesion

**Figure 3:**
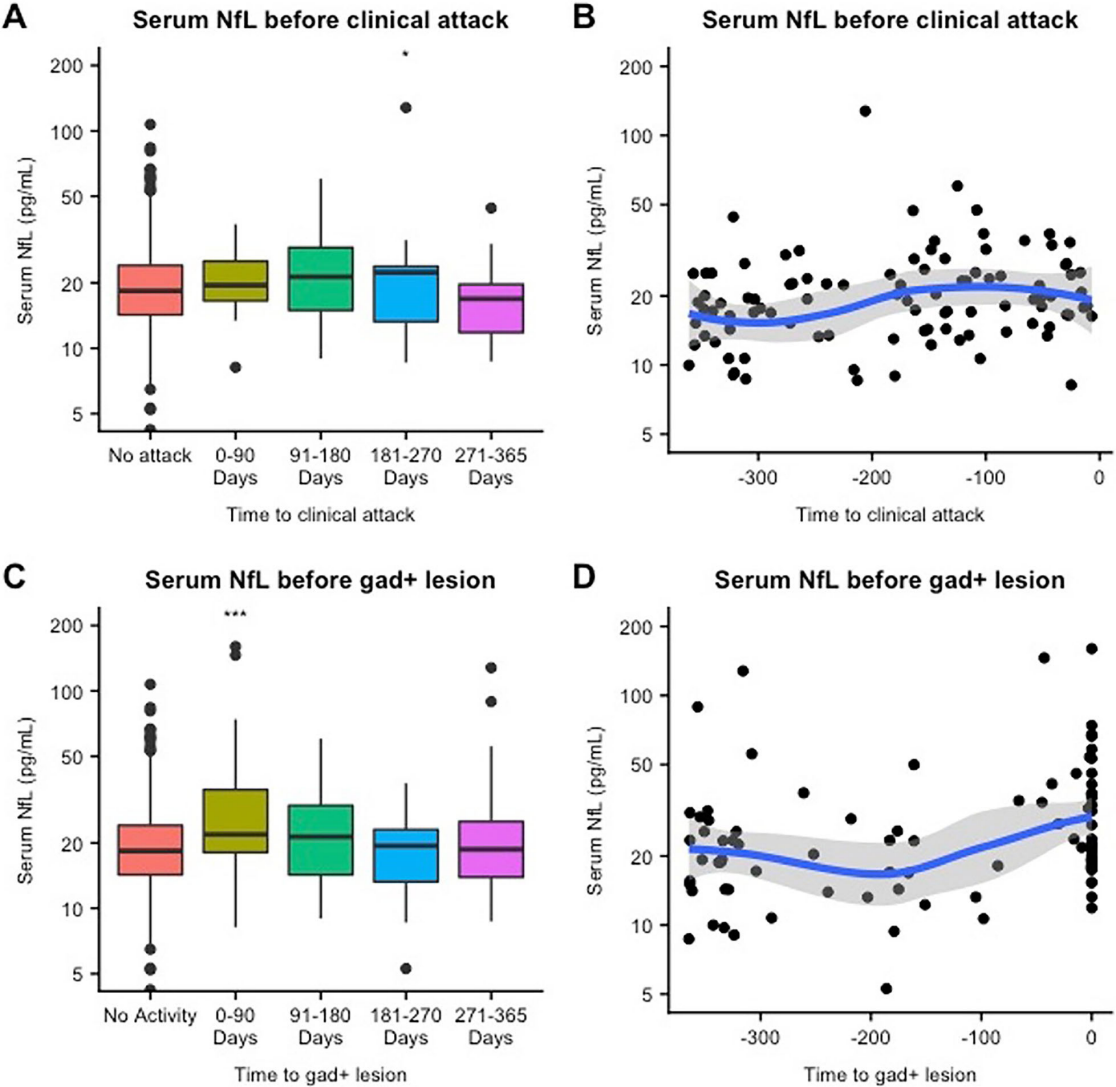
Serum NfL before disease activity. gad + lesion = gadolinium‐enhancing lesion; NfL = neurofilament light chain. **P ≤ 0.001, ***P ≤ 0.0001

We assessed sNfL levels before Gd+ lesions and observed a 32.3% increase in sNfL levels in the 0–90 days time bin prior to a Gd+ lesion compared to remission (95% CI: 17.3%–47.7%, *P* < 0.0001). (Fig. 3C; Table 4) Most serum samples in this time bin were taken a few days prior to the Gd+ lesion. (Fig. 3D) As in the analysis of sNfL levels after Gd+ lesions, this finding was replicated in the combined analysis of clinical relapses and Gd+ lesions. We observed that recent Gd+ lesions were associated with a significant elevation in all groups, that is, in the absence of clinical relapses (estimated percentage increase = 41.9%, 95% CI: 22.1%–64.9%, *P* < 0.0001), with recent clinical relapses (estimated percentage increase = 49.2%, 95% CI: 7.2%–107.5%, *P* = 0.02), and with remote clinical relapses (estimated percentage increase = 180%, 95% CI: 99.4%–289%, *P* < 0.0001). (Table 5).

**Table 4:** sNfL dynamics before disease activity in 3‐month increments.

Time bins	Clinical relapse				MRI Gd+ lesion (gadolinium‐enhancing)			
	Unadjusted^2^		Adjusted^1^, ^2^		Unadjusted^2^		Adjusted^1^, ^2^	
Group	Delta sNfL	*P* value*	Delta sNfL	*P* value*	Delta sNfL	*P* value*	Delta sNfL	*P* value*
No disease activity (*n* = 444)	Ref.	–	Ref.	–	Ref.	–	Ref.	–
0–90 days (clinical: *n* = 26; MRI: *n* = 64)	0.7% (–14.8%–18.5%)	0.94	–0.1% (–15.6%–18.5%)	0.99	32.3% (18.5%–49.2%)	**<0.0001**	32.3% (17.4%–47.7%)	**<0.0001**
91–180 days (clinical: *n* = 31; MRI: *n* = 32)	5.1% (–9.5%–22.1%)	0.55	5.1% (–10.4–22.1%)	0.56	0.5% (–13.9%–17.4%)	0.95	–0.4% (–14.8%–16.2%)	0.96
181–270 days (clinical: *n* = 16; MRI: *n* = 19)	31% (6.2%–61.6%)	**0.01**	31% (6.2%–61.6%)	**0.01**	–8.6% (–24.4%–11.6%)	0.38	–8.6% (–25.2%–10.5%)	0.36
271–365 days (clinical: *n* = 34; MRI: *n* = 56)	–2% (–14.8%–13.9%)	0.83	–2% (–15.6%–13.9%)	0.79	–2% (–13.1%–10.5%)	0.78	–2% (–13.1%–10.5%)	0.75

*n* = number of samples, sNfL = serum neurofilament light chain.

^1^ Analysis adjusted for age, disease duration, disease‐modifying therapy, sex.

^2^ Results are reported as percentage difference in sNfL, with 95% confidence interval and *P*‐values.

* Significant *P*‐values are reported in bold text.

**Table 5:** sNfL dynamics before disease activity.

Clinical groups	MRI groups	Delta sNfL–unadjusted^2^	Delta sNfL–adjusted^1^, ^2^
No clinical relapse	No Gd+ lesion (*n* = 444)	Ref.	Ref.
	Recent Gd+ lesion^3^ (*n* = 32)	41.9% (22.1%–64.9%) ***P* < 0.0001** *	41.9% (22.1%–64.9%) ***P* < 0.0001** *
	Remote Gd+ lesion^4^ (*n* = 32)	–2% (–16.5%–13.9%) *P* = 0.77	–3% (–17.3%–13.9%) *P* = 0.70
Recent clinical relapse^5^	No Gd+ lesion (*n* = 20)	–8.6% (–24.4%–9.4%) *P* = 0.32	–9.5% (–25.2%–9.4%) *P* = 0.29
	Recent Gd+ lesion^3^ (*n* = 6)	49.2% (7.3%–107.5%) *P* = **0.02**	47.7% (5.1%–105.4%) *P* = **0.02**
Remote clinical relapse^6^	No Gd+ lesion (*n* = 62)	–5.8% (–24.4%–9.4%) *P* = 0.28	–5.8% (–16.5%–5.1%) *P* = 0.27
	Recent Gd+ lesion^3^ (*n* = 6)	180.1% (99.4%–289.6%) ***P* < 0.0001** *	180.1% (99.4%–289.6%) ***P* < 0.0001** *
	Remote Gd+ lesion^4^ (*n* = 13)	8.3% (–13.1%–36.3%) *P* = 0.47	7.3% (–13.9%–35%) *P* = 0.52

*n* = number of samples; sNfL, serum neurofilament light chain.

^1^ Analysis adjusted for age, disease duration, disease‐modifying therapy, sex.

^2^ Results are reported as percentage difference in sNfL, with 95% confidence interval and *P*‐values.

^3^ Recent Gd+ lesion = serum sample within 0–90 before Gd+ lesion.

^4^ Remote Gd+ lesion = serum sample within 91–365 before Gd+ lesion.

^5^ Recent clinical relapse = serum sample within 0–90 before clinical relapse.

^6^ Remote clinical relapse = serum sample within 91–365 before clinical relapse.

* Significant *P*‐values are reported in bold text.

### sNfL association with relapse severity and location

To analyze the association between sNfL levels and severity and location, we stratified serum samples according to the time after/before the clinical relapse. We divided serum samples into recent (i.e. within 90 days of sample collection) and remote (i.e. between 91 and 365 days of sample collection) groups. In terms of clinical relapse severity, we observed a significant elevation in sNfL after mild and severe relapses (mild: 24.6%, 95% CI: 4.1%–50.7% *P* = 0.02; severe: 39%, 95% CI: 5%–82%, *P* = 0.02). (Table S1) When we analyzed sNfL levels after a clinical relapse by location, only recent spinal cord relapses were associated with a significant percentage increase in sNfL (estimated increase: 23.4%, 95% CI: 3%–47.7%, *P* = 0.03); recent relapses localized to the brainstem and cerebellum were associated with a percentage increase in sNfL trending toward significance (estimated increase: 28.4%, 95% CI: –1%–64.9%, *P* = 0.06). Other localizations were not associated with a significant elevation in sNfL compared to patients in remission. (Table S2).

We replicated this analysis in serum samples taken before a clinical relapse, we failed to find any significant difference in sNfL levels according to the clinical severity before the clinical relapses. (Table S3) When we analyzed sNfL levels by location after the clinical relapse, only remote cerebral relapses were associated with a significant difference in terms of sNfL levels (estimated increase = 80.4%, 95% CI: 19.7%–171.8%, *P* = 0.006). (Table S4) This group had a very small sample size (four samples), which may affect the interpretability of this outcome. Due to the small sample sizes, we decided not to parse the groups according to the presence of Gd+ lesions.

## Discussion

The main goal of this study was to assess the role of sNfL as a biomarker of clinical relapses and Gd+ lesions. To test this, we examined the average sNfL levels up to 1 year before and after these events and in remission. Our results show that sNfL levels are elevated within a 3‐month window after a Gd+ lesion. Also, we found significantly elevated sNfL levels within 3 months of a clinical relapse, however, this finding was only seen when clinical relapses were associated with Gd+ lesions. In an attempt to clarify the source of sNfL level heterogeneity after a clinical relapse, we assessed the role of relapse severity and location. We observed an association between spinal cord relapses and sNfL levels, which has important clinical implications, as spinal lesions are associated with more severe disease outcomes. Our analyses did not discriminate relapse location and severity according to the presence of Gd+ lesions due to small sample sizes. It would be interesting to include the presence, size, and location of Gd+ lesions in future analyses.

Our study aligns with results from previous studies where sNfL was elevated between 1 and 4 months after new Gd+ lesions.^24^, ^28^, ^32^ It should be noted that we did not find an elevation in sNfL before clinical relapses. Several prior studies showed that higher baseline sNfL levels were associated with future disease activity in terms of clinical relapses and/or accrual in T2 lesion load.^32^, ^33^, ^34^ Nonetheless, these studies differ significantly in their design from ours—they employed long follow‐up times and looked at relapse rates or T2 lesion loads over this time frame. On the other hand, our study addressed whether sNfL was associated with clinical relapses during the 3 months immediately following sample collection.

The strengths of our study include a real‐world patient cohort with a longitudinal follow‐up at a tertiary‐level MS center. An additional strength is longitudinal MRI data, which allowed us to investigate inflammatory activity, such as Gd+ lesions. This patient population is an ideal test for the validity of a biomarker poised to enter clinical practice.

Our study has some limitations, including the lack of CSF NfL levels, which were previously recognized as the gold standard for the measurement of this biomarker. However, a large number of studies have demonstrated a high degree of correlation between CSF and serum NfL levels. Furthermore, serum samples would likely be the tissue of choice for NfL measurement in a clinical setting.^24^, ^35^, ^36^ A final limitation of this study relates to the clinical characteristics of our cohort (e.g., short disease duration and low disability burden) and the low sample size for some Gd+ and clinical relapse time bins. As such, the conclusions of our study may not be completely generalizable to all MS cohorts.

In conclusion, our study confirms that sNfL is associated with Gd+ lesions. Furthermore, sNfL seems to have potential in discriminating between clinical relapses with Gd+ lesions and clinical relapses without Gd+ lesions. One possibility is that clinical relapses without Gd+ lesions may be pseudorelapses or unrelated physical symptoms. Future research is needed to clarify this point. This is a suggestive finding, which further confirms that sNfL correlates with acute CNS inflammation and Gd+ lesions. However, sNfL levels were shown to be subject to a great degree of interindividual and intraindividual variability, which may impair or even invalidate the clinical applications of this biomarker. Therefore, efforts need to be made to address all the possible confounders at play.

## Conflict of Interest

Rosso received support from Verily Life Sciences and Biogen. Gonzalez received research support from Verily Life Sciences. Healy was on the Biogen Worldwide Medical Biostatistics Multiple Sclerosis Advisory Board and received grant support from Genzyme, Merck Serono, and Novartis. Paul: nothing to disclose. Saxena received support from Verily Life Sciences and Biogen. Bjornevik: nothing to disclose. Kuhle received and exclusively used for research support: consulting fees from Biogen, Novartis, Protagen AG, Roche, and Teva; speaker fees from the Swiss MS Society, Biogen, Genzyme, Merck, Novartis, Roche; travel expenses from Merck Serono, Novartis, and Roche; and grants from the ECTRIMS Research Fellowship Programme, University of Basel, Swiss MS Society, Swiss National Research Foundation (320030_160221), Bayer, Biogen, Genzyme, Merck, Novartis, and Roche. Benkert. reports no conflicts of interest. Leppert is an employee of Novartis Pharma AG. Guttmann has received research funding from Sanofi, the National Multiple Sclerosis Society, and the International Progressive Multiple Sclerosis Alliance, the U.S. Office for Naval Research, as well as travel support from Roche Pharmaceuticals; Guttmann owns stock in Roche, Novartis, GSK, Alnylam, Protalix Biotherapeutics, Arrowhead Pharmaceuticals, Cocrystal Pharma, Sangamo Therapeutics. Bakshi has received consulting fees from Bayer, Biogen, Celgene, EMD Serono, Genentech, Guerbet, Sanofi‐Genzyme, and Shire and research support from EMDSerono and Sanofi‐Genzyme. Weiner reports grants from National Institutes of Health, grants from National Multiple Sclerosis Society, Verily Life Sciences, EMD Serono, Biogen, Teva Pharmaceuticals, Sanofi, grants from Novartis, and grants and personal fees from Genentech, Inc, and Tilos Therapeutics, personal fees from Tiziana Life Sciences, IM Therapeutics, MedDay Pharmaceuticals, and vTv Therapeutics, outside the submitted work. Chitnis received personal compensation for advisory board/consulting for Biogen‐Idec, Merck Serono, Novartis, Sanofi, Bayer, Celgene, Alexion and received financial support for research activities from Merck Serono and Novartis Pharmaceuticals. This study was funded in part by EMD Serono.

## Author Contribution

MR – Conception and design of the study, acquisition and analysis of data, drafting a significant portion of manuscript or figures. CTG – Conception and design of the study, acquisition and analysis of data, drafting a significant portion of manuscript or figures. BCH – acquisition and analysis of data, drafting a significant portion of manuscript or figures. SS – acquisition and analysis of data. AP – acquisition and analysis of data. KB – acquisition and analysis of data. JK – acquisition and analysis of data. PB – acquisition and analysis of data. DL – acquisition and analysis of data. CG – acquisition and analysis of data. RB – acquisition and analysis of data. HLW – acquisition and analysis of data. TC – Conception and design of the study, acquisition and analysis of data, study funding.

## Supporting information


**Table S1** sNfL after clinical relapse by severity.
**Table S2** sNfL after clinical relapse by location.
**Table S3** sNfL before clinical relapse by severity.
**Table S4** sNfL before clinical relapse by location.

## References

[1] Confavreux C , Vukusic S . Natural history of multiple sclerosis: a unifying concept. Brain 2006;129:606–616. 16415308 10.1093/brain/awl007

[2] Schumacher GA , Beebe G , Kibler RF , et al. Problems of experimental trials of therapy in multiple sclerosis: report by the panel on the evaluation of experimental trials of therapy in multiple sclerosis. Ann N Y Acad Sci 1965;122:552–568. 14313512 10.1111/j.1749-6632.1965.tb20235.x

[3] Avasarala J . Redefining acute relapses in multiple sclerosis: implications for phase 3 clinical trials and treatment algorithms. Innov Clin Neurosci 2017;14:38–40. 28584696 PMC5451037

[4] Meier DS , Weiner HL , Guttmann CR . Time‐series modeling of multiple sclerosis disease activity: a promising window on disease progression and repair potential? Neurotherapeutics 2007;4:485–498. 17599713 10.1016/j.nurt.2007.05.008 PMC7479736

[5] Novotna M , Paz Soldan MM , Abou Zeid N , et al. Poor early relapse recovery affects onset of progressive disease course in multiple sclerosis. Neurology 2015;85:722–729. 26208962 10.1212/WNL.0000000000001856 PMC4553030

[6] Lublin FD , Baier M , Cutter G . Effect of relapses on development of residual deficit in multiple sclerosis. Neurology 2003;61:1528–1532. 14663037 10.1212/01.wnl.0000096175.39831.21

[7] Hirst C , Ingram G , Pearson O , et al. Contribution of relapses to disability in multiple sclerosis. J Neurol 2008;255:280–287. 18204919 10.1007/s00415-008-0743-8

[8] Rosengren LE , Karlsson JE , Karlsson JO , et al. Patients with amyotrophic lateral sclerosis and other neurodegenerative diseases have increased levels of neurofilament protein in CSF. J Neurochem 1996;67:2013–2018. 8863508 10.1046/j.1471-4159.1996.67052013.x

[9] Lycke JN , Karlsson JE , Andersen O , Rosengren LE . Neurofilament protein in cerebrospinal fluid: a potential marker of activity in multiple sclerosis. J Neurol Neurosurg Psychiatry 1998;64:402–404. 9527161 10.1136/jnnp.64.3.402 PMC2170011

[10] Malmestrom C , Haghighi S , Rosengren L , et al. Neurofilament light protein and glial fibrillary acidic protein as biological markers in MS. Neurology 2003;61:1720–1725. 14694036 10.1212/01.wnl.0000098880.19793.b6

[11] Mattsson N , Andreasson U , Zetterberg H , Blennow K . Association of plasma neurofilament light with neurodegeneration in patients with Alzheimer disease. JAMA Neurol 2017;74:557–566. 28346578 10.1001/jamaneurol.2016.6117 PMC5822204

[12] Weston PSJ , Poole T , Ryan NS , et al. Serum neurofilament light in familial Alzheimer disease: a marker of early neurodegeneration. Neurology 2017;89:2167–2175. 29070659 10.1212/WNL.0000000000004667 PMC5696646

[13] Meeter LH , Dopper EG , Jiskoot LC , et al. Neurofilament light chain: a biomarker for genetic frontotemporal dementia. Ann Clin Transl Neurol 2016;3:623–636. 27606344 10.1002/acn3.325 PMC4999594

[14] Shahim P , Zetterberg H , Tegner Y , Blennow K . Serum neurofilament light as a biomarker for mild traumatic brain injury in contact sports. Neurology 2017;88:1788–1794. 28404801 10.1212/WNL.0000000000003912 PMC5419986

[15] Steinacker P , Huss A , Mayer B , et al. Diagnostic and prognostic significance of neurofilament light chain NF‐L, but not progranulin and S100B, in the course of amyotrophic lateral sclerosis: data from the German MND‐net. Amyotroph Lateral Scler Frontotemporal Degener 2017;18:112–119. 27819158 10.1080/21678421.2016.1241279

[16] Barry DM , Stevenson W , Bober BG , et al. Expansion of neurofilament medium C terminus increases axonal diameter independent of increases in conduction velocity or myelin thickness. J Neurosci 2012;32:6209–6219. 22553027 10.1523/JNEUROSCI.0647-12.2012 PMC3363292

[17] Zetterberg H . Neurofilament light: a dynamic cross‐disease fluid biomarker for neurodegeneration. Neuron 2016;91:1–3. 27387643 10.1016/j.neuron.2016.06.030

[18] Khalil M . Are neurofilaments valuable biomarkers for long‐term disease prognostication in MS? Mult Scler 2018;24:1270–1271. 30066596 10.1177/1352458518791518

[19] Gisslen M , Price RW , Andreasson U , et al. Plasma concentration of the neurofilament light protein (NFL) is a biomarker of CNS injury in HIV infection: a cross‐sectional study. EBioMedicine 2016;3:135–140. 26870824 10.1016/j.ebiom.2015.11.036 PMC4739412

[20] Kuhle J , Barro C , Andreasson U , et al. Comparison of three analytical platforms for quantification of the neurofilament light chain in blood samples: ELISA, electrochemiluminescence immunoassay and Simoa. Clin Chem Lab Med 2016;54:1655–1661. 27071153 10.1515/cclm-2015-1195

[21] Disanto G , Barro C , Benkert P , et al. Serum neurofilament light: a biomarker of neuronal damage in multiple sclerosis. Ann Neurol 2017;81:857–870. 28512753 10.1002/ana.24954 PMC5519945

[22] Khalil M , Teunissen CE , Otto M , et al. Neurofilaments as biomarkers in neurological disorders. Nat Rev Neurol 2018;14:577–589. 30171200 10.1038/s41582-018-0058-z

[23] Piehl F , Kockum I , Khademi M , et al. Plasma neurofilament light chain levels in patients with MS switching from injectable therapies to fingolimod. Mult Scler 2018;24:1046–1054. 28627962 10.1177/1352458517715132

[24] Novakova L , Zetterberg H , Sundstrom P , et al. Monitoring disease activity in multiple sclerosis using serum neurofilament light protein. Neurology 2017;89:2230–2237. 29079686 10.1212/WNL.0000000000004683 PMC5705244

[25] Kuhle J , Disanto G , Lorscheider J , et al. Fingolimod and CSF neurofilament light chain levels in relapsing‐remitting multiple sclerosis. Neurology 2015;84:1639–1643. 25809304 10.1212/WNL.0000000000001491 PMC4409586

[26] Kuhle J , Nourbakhsh B , Grant D , et al. Serum neurofilament is associated with progression of brain atrophy and disability in early MS. Neurology 2017;88:826–831. 28148632 10.1212/WNL.0000000000003653 PMC5331872

[27] Mellergard J , Tisell A , Blystad I , et al. Cerebrospinal fluid levels of neurofilament and tau correlate with brain atrophy in natalizumab‐treated multiple sclerosis. Eur J Neurol 2017;24:112–121. 27699930 10.1111/ene.13162

[28] Varhaug KN , Barro C , Bjornevik K , et al. Neurofilament light chain predicts disease activity in relapsing‐remitting MS. Neurol Neuroimmunol Neuroinflamm 2018;5:e422. 29209636 10.1212/NXI.0000000000000422 PMC5707445

[29] Gauthier SA , Glanz BI , Mandel M , Weiner HL . A model for the comprehensive investigation of a chronic autoimmune disease: the multiple sclerosis CLIMB study. Autoimmun Rev 2006;5:532–536. 17027888 10.1016/j.autrev.2006.02.012

[30] Polman CH , Reingold SC , Banwell B , et al. Diagnostic criteria for multiple sclerosis: 2010 revisions to the McDonald criteria. Ann Neurol 2011;69:292–302. 21387374 10.1002/ana.22366 PMC3084507

[31] Chitnis T , Gonzalez C , Healy BC , et al. Neurofilament light chain serum levels correlate with 10‐year MRI outcomes in multiple sclerosis. Ann Clin Transl Neurol 2018;5:1478–1491. 30564615 10.1002/acn3.638 PMC6292183

[32] Barro C , Benkert P , Disanto G , et al. Serum neurofilament as a predictor of disease worsening and brain and spinal cord atrophy in multiple sclerosis. Brain 2018;141:2382–2391. 29860296 10.1093/brain/awy154

[33] Siller N , Kuhle J , Muthuraman M , et al. Serum neurofilament light chain is a biomarker of acute and chronic neuronal damage in early multiple sclerosis. Mult Scler 2019;25:678–686. 29542376 10.1177/1352458518765666

[34] Kuhle J , Kropshofer H , Haering DA , et al. Blood neurofilament light chain as a biomarker of MS disease activity and treatment response. Neurology 2019;92:e1007–e1015. 30737333 10.1212/WNL.0000000000007032 PMC6442011

[35] Kuhle J , Barro C , Disanto G , et al. Serum neurofilament light chain in early relapsing remitting MS is increased and correlates with CSF levels and with MRI measures of disease severity. Mult Scler 2016;22:1550–1559. 26754800 10.1177/1352458515623365

[36] Hakansson I , Tisell A , Cassel P , et al. Neurofilament levels, disease activity and brain volume during follow‐up in multiple sclerosis. J Neuroinflammation 2018;15:209. 30021640 10.1186/s12974-018-1249-7 PMC6052680

