# Supplementary material for: Temporal association of sNfL and gad‐enhancing lesions in multiple sclerosis
Source: Ann Clin Transl Neurol. 2020 May 25;7(6):945–55. doi: 10.1002/acn3.51060 (PMC7318095; doi:10.1002/acn3.51060)
Supplement: Supplementary file 1 — Table S1 sNfL after clinical relapse by severity. Table S2 sNfL after clinical relapse by location. Table S3 sNfL before clinical relapse by severity. Table S4 sNfL before clinical relapse by location. [file ACN3-7-945-s001.docx]

**Supplementary Materials**

**Supplementary Table 1. sNfL after clinical relapse by severity**

| Group | Severity | Delta sNfL– unadjusted | Delta sNfL– adjusted^1^ |
| --- | --- | --- | --- |
| No clinical relapse  (Reference group, n=403) |  | Ref. | Ref. |
| Recent relapse ^3^ | Mild relapse (n=21) | 23.4%  (3%-47.7%.; p=0.03) | 24.6%  (4.1%-50.7%; p=0.02) |
|  | Moderate relapse (n=27) | 13.9%  (–3%-33.6%; p=0.11) | 13.9%  (–3%-33.6%; p=0.12) |
|  | Severe relapse (n=9) | 35%  (3%-78.6%; p=0.03) | 39%  (5%-82%; p=0.02) |
| Remote relapse ^4^ | Mild relapse (n=41) | 7.3%  (–6.8%-22.1%; p=0.34) | 7.3%  (–6.8%-22.1%; p=0.34) |
|  | Moderate relapse (n=28) | 0.4%  (–13.9%-17.4%; p=0.95) | 1%  (–13.1%-18.5%; p=0.90) |
|  | Severe relapse (n=10) | –2%  (–23.7%-27.1%; p=0.90) | 3%  (–21.3%-33.6%; p-0.85) |

Legend: ^1^ Analysis adjusted for age, disease duration, disease-modifying therapy, sex.

^2^ Results are reported as percentage difference in sNfL, with 95% confidence interval and p-values.

^3^ Recent relapse= serum sample within 0 – 90 after clinical relapse.

^4^ Remote relapse= serum sample within 91 - 365 after clinical relapse.

n= number of samples, sNfL= serum neurofilament light chain

**Supplementary Table 2. sNfL after clinical relapse by location**

|  | Delta sNfL– Unadjusted | | Delta sNfL– Adjusted^1^ | |
| --- | --- | --- | --- | --- |
| Location | Recent relapse^3^ | Remote relapse^4^ | Recent relapse^3^ | Remote relapse^4^ |
| Brainstem/cerebellum  (recent- n=10; remote- n=13) | 27.1%  (–2%-63.3%; p=0.07) | 4.1%  (–17.3%-29.7%; p=0.75) | 28.4%  (–1%-64.9%; p=0.06) | 6.2%  (–14.8%-33.6%; p=0.59) |
| Cerebrum  (recent- n=5, remote- n=4) | 33.6%  (–6.8%-89.6%; p=0.11) | 41.9%  (–3.9%-111.7%; p=0.08) | 37.7%  (–3.9%-97.4%; p=0.08) | 46.2%  (–2%-118.1%; p=0.07) |
| Combined location  (recent- n=1, remote- n=3) | 47.7%  (–33%-225.4%; p=0.33) | 53.7%  (–3%-141.1%; p=0.07) | 35%  (–38.1%-197.4%; p=0.45) | 49.2%  (–5.8% -134%; p=0.09) |
| Optic nerve  (recent- n=11, remote- n=19) | –7.7%  (–28.1%-18.5%; p=0.54) | –2%  (–18.9%-19.7%; p=0.86) | –5.8%  (–26.7%-20.9%; p=0.66) | –0.03%  (–18.1%-22.1%; p=0.99) |
| Spinal cord  (recent- n=21, remote- n=34) | 22.1%  (2%-46.2%; p=0.03) | –3.9%  (–16.5%-10.5%; p=0.56) | 23.4%  (3%-47.7%; p=0.03) | –3.9%  (–16.5%-10.5%; p=0.62) |

Legend: ^1^ Analysis adjusted for age, disease duration, disease-modifying therapy, sex.

^2^ Results are reported as percentage difference in sNfL, with 95% confidence interval and p-values.

^3^ Recent relapse= serum sample within 0 – 90 after clinical relapse.

^4^ Remote relapse= serum sample within 91 - 365 after clinical relapse.

^5^ We used patients with ‘no disease activity’ (n=403) as our reference group in these analyses.

n= number of samples, sNfL= serum neurofilament light chain.

**Supplementary Table 3. sNfL before clinical relapse by severity**

| Group | Severity | Delta NfL– unadjusted | Delta NfL– adjusted^1^ |
| --- | --- | --- | --- |
| No clinical relapse  (Reference group) |  | Ref. | Ref. |
| Recent relapse^3^ | Mild relapse  (n=10) | 12.3%  (-12.6%-44.3%; p=0.37) | 13.6%  (-11.7%- 46.2%; p=0.33) |
|  | Moderate relapse  (n=12) | -9.6%  (-28.4%-14.3%; p=0.40) | - 10.2%  (-29.1%- 13.9%; p=0.38) |
|  | Severe relapse  (n=1) | 67%  (-26.3%-278.3%; p=0.22) | 64.4%  (-27.6%- 274.9%; p=0.24) |
| Remote relapse^4^ | Mild relapse  (n=30) | ­–3.5%  (-17.3%-12.6%; p=0.65) | -3.6%  (-17.4%- 12.6%; p=0.65) |
|  | Moderate relapse  (n=37) | 2%  (-11.1%-17.1%; p=0.78) | 2.2%  (-11.1%- 17.4%; p=0.76) |
|  | Severe relapse  (n=10) | -14.6%  (-34.4%-11.1%; p=0.24) | -3.6%  (-33.3%- 13.5%; p=0.31) |

Legend: ^1^ Analysis adjusted for age, disease duration, disease-modifying therapy, sex.

^2^ Results are reported as mean change in serum NfL, with 95% confidence interval.

^3^ Recent relapse= serum sample within 0 – 90 before clinical relapse.

^4^ Remote relapse= serum sample within 91 - 365 before clinical relapse.

n= number of samples, NfL= neurofilament light chain

**Supplementary Table 4. sNfL before clinical relapse by location**

|  | Unadjusted | | Adjusted^1^ | |
| --- | --- | --- | --- | --- |
| Location | Recent relapse^3^ | Remote relapse^4^ | Recent relapse^3^ | Remote relapse^4^ |
| Brainstem/cerebellum  (recent: n=2, remote: n=10) | 9.4%  (–38.7%-95.4%, p=0.76 ) | –3%  (–25.9%-25.9%; p=0.80) | 12.7%  (–36.9%-101.4%; p=0.70) | –3.9%  (–26.7%-24.6%; p=0.74) |
| Cerebrum  (recent: n=2, remote: n=4) | 24.6%  (–29.5%-120.3%, p=0.45) | 78.6%  (18.5%-171.8%; p=0.006) | 23.4%  (–30.2%-118.1%; p=0.47) | 80.4%  (19.7%-171.8%; p=0.006) |
| Combined location  (recent: n=2, remote: n=7) | 27.1%  (–28.1%-124.8%, p=0.42) | –11.3%  (–34.3%-20.9%; p=0.46) | 27.1%  (–27.4%-124.8%; p=0.41) | –13.1%  (–36.2%-18.5%; p=0.38) |
| Optic nerve  (recent: n=4. Remote: n=17) | 22.1%  (–19.7%-85.9%, p=0.36) | 20.9%  (–3%-49.2%; p=0.09) | 22.1%  (–19.7%-85.9%; p=0.35) | 20.9%  (–2%-50.7%; p=0.08) |
| Spinal cord  (recent: n=12, remote: n=27) | –11.3%  (–30.2%-13.9%, p=0.34) | 9.4%  (–6.8%-29.7%; p=0.27) | –12.2%  (–31.6%-12.7%; p=0.30) | 9.4%  (–6.8%-29.7%; p=0.29) |
| Unknown location  (recent: n=4, remote: n=7) | –3.9%  ( –36.9%-47.7%, p=0.86) | –5.8%  (–22.9%-16.2%; p=0.60) | –6.8%  (–38.7%-43.3%; p=0.76) | –3.9%  (–22.9%-18.5%;p=0.68) |

Legend: ^1^ Analysis adjusted for age, disease duration, disease-modifying therapy, sex.

^2^ Results are reported as percentage difference in sNfL, with 95% confidence interval and p-values.

^3^ Recent relapse= serum sample within 0 – 90 before clinical relapse.

^4^ Remote relapse= serum sample within 91 - 365 before clinical relapse.

^5^ We used patients with ‘no disease activity’ (n=444) as our reference group in these analyses.

n= number of samples, sNfL= serum neurofilament light chain
